# Prognostic relevance of persistent haematuria in patients with lupus nephritis

**DOI:** 10.1093/ckj/sfaf348

**Published:** 2025-11-13

**Authors:** Xuan Chen, Lin-Lin Li, Na Li, Pei Wang, Hui-Xia Cao

**Affiliations:** Department of Nephrology, People’s Hospital of Zhengzhou University, Henan Provincial People’s Hospital, Zhengzhou, China; Department of Nephrology, People’s Hospital of Zhengzhou University, Henan Provincial People’s Hospital, Zhengzhou, China; Department of Nephrology, People’s Hospital of Zhengzhou University, Henan Provincial People’s Hospital, Zhengzhou, China; Henan Provincial Key Laboratory of Kidney Disease and Immunology, Zhengzhou, China; Department of Nephrology, People’s Hospital of Zhengzhou University, Henan Provincial People’s Hospital, Zhengzhou, China; Department of Nephrology, People’s Hospital of Zhengzhou University, Henan Provincial People’s Hospital, Zhengzhou, China; Henan Provincial Key Laboratory of Kidney Disease and Immunology, Zhengzhou, China

**Keywords:** haematuria, lupus nephritis, persistent haematuria, prognosis

## Abstract

**Background:**

This study aimed to investigate the significance of persistent haematuria in lupus nephritis (LN).

**Methods:**

A total of 178 patients with LN were enrolled and regularly followed up from December 2016 to September 2022. Follow-up duration was from LN diagnosis to the patient’s latest visit or terminated at the onset of endpoint events if they occurred. Their clinical and laboratory data as well as outcomes were monitored and analysed from the enrolment point until the final visit.

**Results:**

A total of 138 of 178 (77.5%) patients with LN presented with haematuria at the initial diagnosis. During follow-up, 34 patients (19.1%) had no haematuria, 72 (40.4%) patients initially diagnosed but later resolved and 66 (37.1%) exhibited persistent haematuria. Furthermore, patients with persistent haematuria showed higher Systemic Lupus Erythematosus Disease Activity Index scores (12.3 ± 2.9 versus 10.7 ± 3.6; *P* = .002), lower haemoglobin (92.7 ± 25.4 g/l versus 102.0 ± 21.1; *P* = .010), albumin (24.9 ± 6.8 g/l versus 27.5 ± 7.6; *P* = .024) and decreased C3 {median 0.4 g/l [interquartile range (IQR) 0.3–0.5]} versus 0.5 [0.3–0.6]; *P* = .009} and C4 [median 0.1 g/l (IQR 0.0–0.1) versus 0.1 (0–0.1); *P* = .012] and elevated blood urea nitrogen and serum creatinine [median 8.9 mmol/l (IQR 6.7–14.8) versus 5.7 (4.4–8.8) and median 90.0 μmol/l (IQR 64.5–132.3) versus 62.0 (48.0–80.0); all *P* < .001, respectively] compared with those without persistent haematuria (*n* = 106). In time-dependent Cox proportional hazards regression models, persistent haematuria exhibited a significant overall time-averaged effect on composite clinical endpoints, with an adjusted hazard ratio of 3.40 (95% CI 1.06–10.90; *P* = .039). Notably, this risk effect was not fixed but showed an increasing trend over time—at 1 months of follow-up, the risk of adverse outcomes in patients with persistent haematuria had increased to 10.37-fold.

**Conclusions:**

Almost 37.1% of patients exhibited persistent haematuria in LN. Furthermore, persistent haematuria independently predicted adverse outcomes, with significantly higher risks for all-cause mortality and progression to renal replacement therapy.

## GRAPHICAL ABSTRACT

**GRAPHICAL ABSTRACT:**
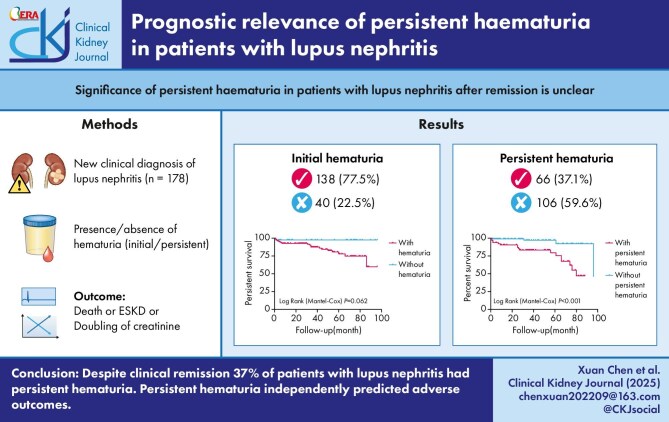

KEY LEARNING POINTS **What was known:**

- Approximately one-third of lupus nephritis (LN) patients who achieve complete clinical remission by conventional criteria still show histopathological activity on follow-up renal biopsies, indicating traditional indicators of remission, such as proteinuria and serological markers, may not fully reflect ongoing renal inflammation.

**This study adds:**

- A total of 37.1% of patients exhibited persistent haematuria in LN. Furthermore, persistent haematuria independently predicted adverse outcomes, with significantly higher risks for all-cause mortality and progression to renal replacement therapy.

**Potential impact:**

- Continuous monitoring of haematuria in clinical practice, along with targeted intervention studies, is essential to improve the long-term prognosis for patients with LN.

## INTRODUCTION

Systemic lupus erythematosus (SLE) is an autoimmune disease characterized by multisystem involvement, with ≈30–60% of patients developing lupus nephritis (LN) during disease progression [1]. While modern immunosuppressive therapy has significantly improved short-term outcomes, studies demonstrate that nearly10–50% of LN patients progress to chronic kidney disease (CKD), with long-term follow-up data revealing 5-year and 10-year end-stage renal disease (ESRD) incidence rates of 3–11% and 16–19%, respectively, in these patients [2, 3]. The development of CKD and ESRD not only substantially increases mortality risk but also exacerbates complication burden, presenting significant challenges for clinical management [4].

Haematuria serves as a common diagnostic indicator for identifying patients with proliferative glomerulonephritis. Moreover, emerging evidence indicates that persistent haematuria independently predicts critical prognostic value in various forms of glomerulonephritis, including immunoglobulin A nephropathy (IgAN), anti-neutrophil cytoplasmic antibody (ANCA)-associated vasculitis (AAV) and primary membranous nephropathy (PMN) [5–8]. Similar findings have been observed in LN, with acanthocytes identified as the optimal biomarker for predicting proliferative LN [area under the curve (AUC) = 0.743] [9]. Moreover, acanthocyte levels also exhibited strong correlations with both the activity index and chronicity index in renal biopsies. Additionally, another study established that a dysmorphic erythrocyte proportion >40% in urinary sediment effectively distinguished proliferative from non-proliferative LN, demonstrating high sensitivity (91.2%) and specificity (79.17%) [10]. Notably, while the majority of patients manifesting isolated haematuria and/or pyuria exhibit demonstrable renal or extrarenal SLE disease activity during initial clinical assessment or within the peri-evaluation period, active urinary sediment components (particularly haematuria) lack sufficient sensitivity and specificity to serve as reliable biomarkers for either LN flare or its severity, warranting further investigation to elucidate their definitive clinical relevance [11, 12].

In current clinical practice, LN monitoring predominantly focuses on proteinuria and renal function. However, a prospective cohort study revealed that even among LN patients achieving sustained clinical remission, 55.3% experienced disease recurrence after a median follow-up of 3.6 years while the role of persistent haematuria in this process remains incompletely elucidated [13]. Accordingly, this study aims to investigate, within a large Chinese cohort of LN patients, whether persistent microscopic haematuria during follow-up can independently predict adverse outcomes—including death, ESRD or doubling of serum creatinine—in initially diagnosed LN patients who have achieved clinical remission with stable conventional indicators such as proteinuria and renal function. The objective is to provide evidence for optimizing long-term LN monitoring and risk stratification strategies.

## MATERIALS AND METHODS

### Patients

**Figure 1:**
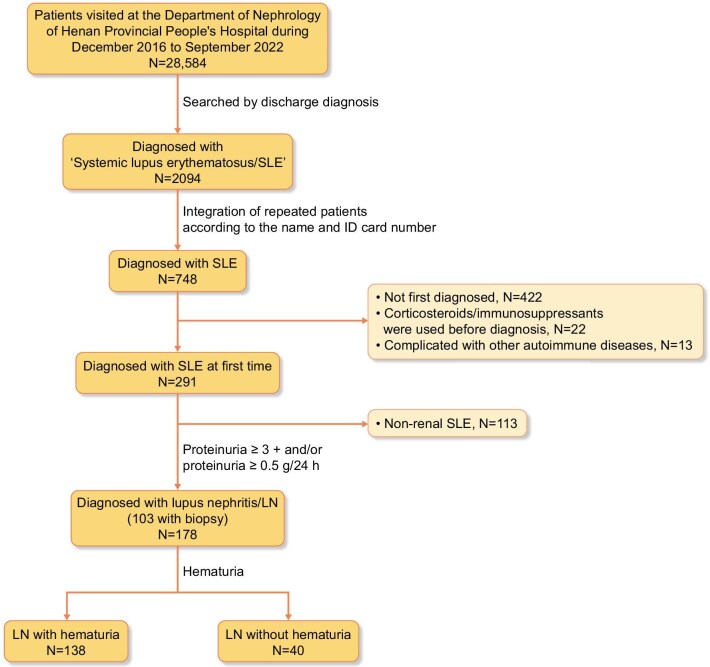
Enrolment of LN patients from the Henan Provincial People’s Hospital.

This is a retrospective study of patients with SLE/LN; the flow chart is shown in Fig. 1. A total of 748 patients with SLE were enrolled in Henan Provincial People’s Hospital from December 2016 to September 2022. They all fulfilled the 2019 European League Against Rheumatism/American College of Rheumatology classification criteria for SLE [14]. Considering the influences of glucocorticoids, immunosuppressants and other drugs, 422 patients were previously diagnosed with LN, 22 had medical treatment before diagnosis and 13 cases were complicated with other autoimmune diseases (e.g. rheumatoid arthritis, IgA nephritis, ANCA-associated vasculitis) were excluded. Among the initially identified patients, 291 were confirmed as newly diagnosed with SLE. Following the exclusion of 113 patients with non-renal SLE, 178 patients with complete clinical and laboratory data who were newly diagnosed with LN—defined by proteinuria ≥3+ and/or proteinuria ≥0.5 g/24 h—were ultimately enrolled, among which 103 cases had a renal biopsy.

The research was in compliance of the Declaration of Helsinki and approved by the local ethical committees of Henan Provincial People’s Hospital [no. 2019(12)]. Written informed consents were obtained from all enrolled individuals.

### Clinical and pathological index

The following data were collected and analysed: age, gender, fever (non-infectious), malar rash, photosensitivity, oral ulcer, alopecia, arthralgia, serositis, neurologic disorder, anaemia, acute kidney injury (AKI), nephrotic syndrome, leukocytopenia, thrombocytopenia, haematuria and leukocyturia. Clinical SLE disease activity was assessed using the Systemic Lupus Erythematosus Disease Activity Index 2000 (SLEDAI-2K) [15]. Serum anti-nuclear antibodies (ANAs), anti-double-stranded DNA (dsDNA) antibodies and serum C3 and C4 levels were determined at every visit during follow-up. The estimated glomerular filtration rate (eGFR) was obtained using the Chronic Kidney Disease Epidemiology Collaboration two-level race equation. Treatment regimens, including both induction and maintenance therapies, were collected for a cohort of 178 patients with LN. Haematuria was defined as red blood cells (RBCs) >5 per high-power field (HPF). Persistent haematuria was defined as the presence of >5 RBCs per HPF in urinary sediment on two or more consecutive follow-up visits during the observation period (from LN diagnosis to the last follow-up or endpoint event onset). AKI was diagnosed in accordance with the criteria outlined in the 2012 Clinical Practice Guideline for Acute Kidney Injury issued by the Kidney Disease: Improving Global Outcomes (KDIGO) [16]. Per this guideline, AKI is identified if any of the following three conditions are satisfied within a 7-day window: a rapid increase in serum creatinine (SCr) of ≥0.3 mg/dl (equivalent to ≥26.5 μmol/l) occurring within 48 h, an elevation of SCr to ≥1.5 times the patient’s baseline SCr level or a reduction in urine output to <0.5 ml/kg of body weight/h, sustained for at least 6 h.

The renal biopsy specimens were examined by light microscopy and immunofluorescence. They were all classified according to the International Society of Nephrology and Renal Pathology Society 2018 classification. The pathological parameters, including activity indices and chronicity indices, were assessed by semi-quantitative scoring as previously [17]. In this study, pathological types were categorized into two groups: proliferative lupus nephritis, comprising classes III, IV, III + V and IV + V; and non-proliferative LN, including class II and pure class V (without concurrent class III or IV lesions).

The patients were regularly followed up in outpatient lupus clinics specified for LN patients or by telephone. The primary endpoint was defined as death (for any causes), the secondary endpoint was defined as ESRD or doubling of SCr and a composite endpoint consisted of the primary and secondary endpoints.

### Statistical analysis

SPSS 26.0 (IBM, Armonk, NY, USA) was used for statistical analysis. Quantitative data were expressed as mean ± standard deviation (SD) or median [interquartile range (IQR)]. For continuous variables, the unpaired *t*-test and Mann–Whitney U test were used for comparison of continuous data. For categorical variables, the chi-squared or Fisher’s exact test was used. Univariable and multivariate logistic regression analyses were performed to identify independent determinants of persistent haematuria during disease flares. Kaplan–Meier curves were used to analyse patients’ prognoses. Survival analysis using univariate and multivariable Cox regression was performed to test the association and a composite event. *P*-values <.05 were considered statistically significant. Confidence intervals (CIs) included 95% of predicted values. All Cox models were verified for proportional hazards (PH) assumptions using Schoenfeld residuals. Standard Cox models were applied when PH assumptions were satisfied; otherwise, non-compliant variables were incorporated as stratification factors or time-dependent Cox models were employed. Analyses included the full cohort (*n* = 178), adjusted for age, baseline creatinine, proteinuria, C3, haemoglobin, C-reactive protein (CRP), non-infectious leukocyturia, AKI and immunosuppressive regimens; and the biopsy-confirmed subgroup (*n* = 103), adjusted for age, baseline SCr, proteinuria, low C3 and pathological classification.

## RESULTS

### Baseline characteristics of the overall cohort (***n*** = 178)

A total of 178 patients with LN were enrolled and regularly followed up. The clinical and laboratory characteristics are presented in Table 1. The cohort exhibited a male:female ratio of 1:4 (35 males:143 females) and a predominance of females (80.3%). The mean age of the participants was 37 ± 15 years. The use of immunosuppressants among the 178 patients with LN, including both induction and maintenance treatment regimens, is presented in Table 3. Among these patients, 103 cases underwent a kidney biopsy and histopathological classifications revealed class II [8 (7.8%)], class III [4 (3.9%)], class IV [26 (25.2%)], class V [35 (34.0%)], class III + V [14 (13.6%)] and class IV + V [16 (15.5%)]. The median follow-up time was 34.0 months (IQR 7.8–59.3). During the disease course, 9 patients died and 12 patients reached the secondary endpoint, including 6 with ESRD and 6 with doubling of SCr.

**Table 1:** Clinical and laboratory characteristics: total cohort of 178 LN patients and subgroups with and without initial haematuria.

Characteristics	LN (*n* = 178)	LN without haematuria (*n* = 40)	LN with haematuria (*n* = 138)	*P*-value
Clinical evaluation				
Sex (male/female), *n*	35/143	6/34	29/109	.399
Age (years), mean ± SD	37 ± 15	36.8 ± 13.9	37.1 ± 14.8	.907
SLEDAI (non-renal), median (IQR)	2.0 (0–4.0)	2.0 (1.0–4.0)	2.0 (0–4.0)	.638
SLEDAI, mean ± SD	11.2 ± 3.5	7.9 ± 3.2	12.1 ± 2.9	<.001
Fever (non-infectious), *n* (%)	47 (26)	12 (30)	35 (25)	.558
Malar rash, *n* (%)	49 (28)	13 (33)	36 (26)	.424
Photosensitivity, *n* (%)	25 (14)	7 (18)	18 (13)	.475
Oral ulcer, *n* (%)	3 (2)	1 (3)	2 (1)	.536
Alopecia, *n* (%)	6 (3)	2 (5)	4 (3)	.618
Arthralgia, n (%)	38 (21)	11 (28)	27 (20)	.281
Serositis, *n* (%)	32 (18)	4 (10)	28 (20)	.136
Neurologic disorder, *n* (%)	5 (3)	2 (5)	4 (3)	>.999
AKI, *n* (%)	32 (18)	2 (5)	30 (22)	.015
Laboratory assessment				
Proteinuria, *n* (%)	178 (100)	36 (90)	132 (96)	.235
Leukocyturia (non-infection), *n* (%)	59 (33)	7 (18)	52 (38)	.017
Haemoglobin (g/l), mean ± SD	98.6 ± 23.1	107.7 ± 19.5	97.7 ± 24.1	.334
Platelets, mean ± SD	162.9 ± 80.1	191.7 ± 76.2	154.4 ± 79.5	.010
Total protein (g/l), mean ± SD	56.5 ± 13.3	60.2 ± 12.6	55.4 ± 13.4	.043
Albumin (g/l), mean ± SD	26.5 ± 7.4	29.8 ± 6.7	25.6 ± 7.4	.002
Globulin (g/l), mean ± SD	29.7 ± 8.1	31.4 ± 6.3	29.2 ± 8.5	.124
BUN (mmol/l), median (IQR)	6.8 (4.8–10.4)	4.9 (3.5–6.1)	7.4 (5.3–11.2)	<.001
SCr (μmol/l), median (IQR)	69.5 (51.0–99.0)	51.5 (42.3–58.8)	75.5 (57.8–110.3)	<.001
Urine protein (g/24 h), median (IQR)	2.8 (1.3–5.8)	1.5 (0.7–3.8)	3.1 (1.6–6.0)	.002
IgA (g/l), median (IQR)	2.7 (1.9–3.5)	2.7 (2.0–3.9)	2.7 (1.9–3.5)	.428
IgG (g/l), mean ± SD	14.3 ± 6.8	14.8 ± 5.8	14.1 ± 7.1	.560
IgM (g/l), median (IQR)	1.1 (0.7–1.5)	1.04 (0.62–1.54)	1.05 (0.68–1.47)	.911
C3 (g/l), median (IQR)	0.4 (0.3–0.6)	0.6 (0.4–0.8)	0.4 (0.3–0.6)	<.001
C4 (g/l), median (IQR)	0.1 (0–0.1)	0.1 (0–0.2)	0.1 (0–0.1)	.018
CRP (mg/l), mean ± SD	2.6 (0.5–6.7)	2.2 (0.5–7.5)	2.7 (0.5–6.0)	.934
ESR (mm/h), mean ± SD	36.3 ± 23.2	37.1 ± 18.37	36.1 ± 24.6	.873
Anti-U1RNP antibody positive, *n* (%)	82 (46.1)	15 (38)	67 (49)	.276
Anti-Smith antibody positive, *n* (%)	66 (37)	14 (35)	52 (38)	.701
Anti-dsDNA antibody positive, *n* (%)	46 (26)	7 (18)	39 (28)	.266
Anti-SSA antibody positive, *n* (%)	88 (49.4)	22 (55)	66 (48)	.475
Anti-SSB antibody positive, *n* (%)	25 (14)	5 (13)	20 (15)	.705
Adverse outcomes, *n* (%)	21 (11.8)	20 (14.5)	1 (2.5)	.049

ESR: erythrocyte sedimentation rate.

### Comparative analysis of haematuria-associated clinical features

#### Clinical and laboratory characteristics between patients with and without initial haematuria

All participants were categorized into two groups based on the presence of haematuria (positive versus negative) at the initial diagnosis. A total of 138 patients were classified into the haematuria group and 40 were classified into the non-haematuria group (shown in Fig. 1). Compared with patients without haematuria at the initial diagnosis, those with haematuria exhibited higher SLEDAI scores (12.1 ± 2.9 versus 7.9 ± 3.2; *P* < .001), a significantly higher prevalence of AKI (22% versus 5%; *P* = .015), lower levels of C3 [median 0.4 g/l (IQR 0.3–0.6) versus 0.6 (0.4–0.8); *P* < .001] and C4 [median 0.1 g/l (IQR 0.0–0.1) versus 0.1 (0.0–0.2); *P* = .018], as well as decreased platelets, total protein and albumin [154.4 ± 79.5 versus 191.7 ± 76.2, 55.4 ± 13.3 g/l versus 60.2 ± 12.6, 25.6 ± 7.4 g/l versus 29.8 ± 6.7; *P* =.010, .043 and .002, respectively) (shown in Table 1).

Furthermore, patients with haematuria demonstrated a significantly higher prevalence of proteinuria and leukocyturia compared with those without haematuria (96% versus 90%, *P* = .235; 38% versus 18%, *P* = .017, respectively). They also had significantly higher values of 24-h urine protein [3.1 g/24 h (IQR 1.6–6.0) versus 1.5 (0.7–3.8); *P* = .002) and SCr (75.5 μmol/l (IQR 57.8–110.3) versus 51.5 (42.3–58.8); *P* < .001). There were no differences in other clinical and laboratory features (detailed in Table 1).

#### Clinical and laboratory characteristics between patients with and without persistent haematuria

A total of 34 of the 178 (19%) patients did not have haematuria throughout the entire course, 72 (40%) patients initially presented with haematuria but later resolved, 66 (37%) exhibited persistent haematuria and 6 (3%) were initially negative for haematuria but developed haematuria later. We classified the patients who had negative haematuria or converted to negative during follow-up as one group and those with persistent haematuria as another group (shown in Table 2). The comparison of treatment regimens between the persistent and non-persistent haematuria groups is presented in Table 3. The results showed a non-significant trend toward a higher initial haematuria rate in the cyclophosphamide (CYC) group than in the non-CYC group (84.2% versus 72.5%; *P* = .065). In contrast, the CYC group had slightly lower rates of persistent haematuria (32.9% versus 40.2%; *P* = .318) and endpoint events (12.7% versus 10.5%; *P* = .650), with no statistical significance for these differences. Compared with 106 patients without persistent haematuria, the 66 patients with persistent haematuria exhibited higher SLEDAI scores (12.3 ± 2.9 versus 10.7 ± 3.6; *P* = .002), significantly lower levels of haemoglobin (92.7 ± 25.4 g/l versus 102.0 ± 21.1; *P* = .010), albumin (24.9 ± 6.8 g/l versus 27.5 ± 7.6; *P* = .024), C3 [0.4 g/l (IQR 0.3–0.5) versus 0.5 (0.3–0.6); *P* = .009] and C4 [0.1 g/l (IQR 0.0–0.1) versus 0.1 (0.0–0.1); *P* = .012], as well as higher levels of blood urea nitrogen [BUN; 8.9 mmol/l (IQR 6.7–14.8) versus 5.7 (4.4–8.8); *P* < .001] and SCr [90.0 μmol/l (IQR 64.5–132.3) versus 62.0 (48.0–80.0); *P* < .001) (displayed in Table 2).

**Table 2:** Clinical and laboratory characteristics of patients with and without persistent haematuria.

Characteristics	LN with persistent haematuria (*n* = 66)	LN without persistent haematuria (*n* = 106)	*P*-value
Clinical evaluation			
Sex (male/female), *n*	17/49	17/89	.120
Age (years), mean ± SD	38.26 ± 17.15	36.78 ± 12.96	.550
SLEDAI (non-renal), median (IQR)	2.0 (0–4.0)	2.0 (0–4.0)	.511
SLEDAI, mean ± SD	12.3 ± 2.9	10.7 ± 3.6	.002
Fever (non-infectious), *n* (%)	13 (20)	30 (28)	.205
Malar rash, *n* (%)	15 (23)	31 (29)	.348
Photosensitivity, *n* (%)	9 (14)	14 (13)	.936
Oral ulcer, *n* (%)	0 (0)	3 (3)	.286
Alopecia, *n* (%)	2 (3)	4 (4)	>.999
Arthralgia, *n* (%)	12 (18)	24 (23)	.484
Serositis, *n* (%)	16 (24)	16 (15)	.134
Neurologic disorder, *n* (%)	2 (3)	3 (3)	>.999
AKI, *n* (%)	16 (24)	16 (15)	.134
Laboratory assessment			
Haemoglobin (g/l), mean ± SD	92.7 ± 25.4	102.0 ± 21.1	.010
Platelets, mean ± SD	146.2 ± 75.2	170.0 ± 81.4	.057
Total protein (g/l), mean ± SD	54.2 ± 14.1	57.7 ± 12.9	.098
Albumin (g/l), mean ± SD	24.9 ± 6.8	27.5 ± 7.6	.024
Globulin (g/l), mean ± SD	28.1 ± 8.9	30.6 ± 7.6	.051
BUN (mmol/l), median (IQR)	8.9 (6.7–14.8)	5.7 (4.4–8.8)	<.001
SCr (μmol/l), median (IQR)	90.0 (64.5–132.3)	62.0 (48.0,80.0)	<.001
Urine protein (g/24 h), median (IQR)	3.44 (2.02–7.23)	2.60 (0.95–5.60)	.008
IgA (g/l), median (IQR)	2.5 (1.8–3.3)	2.7 (2.0–3.7)	.267
IgG (g/l), mean ± SD	13.8 ± 6.8	14.5 ± 6.94	.518
IgM (g/l), median (IQR)	1.1 (0.7–1.4)	1.0 (0.7–1.5)	.582
C3 (g/l), median (IQR)	0.4 (0.3–0.5)	0.5 (0.3–0.6)	.009
C4 (g/l), median (IQR)	0.1 (0–0.1)	0.1 (0–0.1)	.122
Anti-Smith antibody positive, *n* (%)	20 (30)	45 (43)	.108
Anti-dsDNA antibody positive, *n* (%)	20 (30)	25 (24)	.290
Adverse outcomes, *n* (%)	17 (25.8)	4 (3.6)	<.001

ESR: erythrocyte sedimentation rate.

**Table 3:** Treatment regimens and cyclophosphamide-associated outcomes in 178 LN patients: total cohort and subgroups with and without persistent haematuria.

Treatments	LN (*n* = 178)	LN with persistent haematuria (*n* = 66)	LN without persistent haematuria (*n* = 106)	CYC group (*n* = 76) versus non-CYC group (*n* = 102), *n* (%); *P*-value
Induction therapy				
G + HCQ, *n* (%)	32 (18.0)	7 (10.6)	24 (22.6)	
G + HCQ + MMF, *n* (%)	62 (34.8)	30 (45.5)	30 (28.3)	
G + HCQ + CYC, *n* (%)	76 (42.7)	25 (37.9)	49 (46.2)	
G + HCQ + TAC, *n* (%)	5 (2.8)	2 (3)	3 (2.8)	
G + HCQ + MMF + TAC, *n* (%)	3 (1.7)	2 (3)	0 (0)	
Maintenance therapy				
G + HCQ, *n* (%)	32 (18.0)	7 (10.6)	24 (22.6)	
G + HCQ + MMF, *n* (%)	146 (82)	59 (89.4)	82 (77.4)	
CYC-associated outcomes				
Initial haematuria, *n* (%)	138 (77.5)	66 (100)	72 (67.9)	64 (84.2) versus 74 (72.5); χ^2^ = 3.40; *P* = .065
Persistent haematuria, *n* (%)	66 (37.1)	66 (100)	0 (0)	25 (32.9) versus 41 (40.2); χ^2^ = 1.00, *P* = .318
Adverse outcomes, *n* (%)	21 (11.8)	17 (25.8)	4 (3.6)	8 (10.5) versus 13 (12.7); χ^2^ = 0.21, *P* = .650

G: glucocorticoids; HCQ: hydroxychloroquine; MMF: mycophenolate mofetil; CYC: cyclophosphamide; TAC: tacrolimus.

#### Binary logistic regression for factors associated with persistent haematuria at onset (***n*** = 178)

Binary logistic regression analysis was conducted to identify significant predictors of persistent haematuria during disease onset. Initial evaluation of nine candidate variables revealed five independent risk factors that were retained in the final predictive model: SLEDAI score, C3 level, SCr level, presence of AKI and non-infectious leukocyturia. Notably, each 0.2 g/l reduction in C3 was associated with an 16.9% increased risk of persistent haematuria [adjusted odds ratio (aOR) 0.16 (95% CI 0.03–0.90), *P* = .037]. Additionally, every 1 mg/dl increase in SCr corresponded to a 62% higher risk of persistent haematuria [aOR 1.62 (95% CI 1.04–2.54); *P* = .035) (displayed in Table 4).

**Table 4:** Binary logistic regression for factors associated with persistent haematuria at onset (*n* = 178).

	Univariable logistic regression		Multivariable logistic regression	
Variables	OR (95% CI)	*P*-value	aOR (95% CI)	*P*-value
Sex (male versus female)	0.55 (0.26–1.17)	.119		
Age (per 1 year)	1.01 (0.99–1.03)	.400		
SLEDAI	1.17 (1.06–1.28)	.001	1.09 (0.97–1.23)	.137
C3	0.09 (0.02–0.42)	.002	0.16 (0.03–0.90)	.037
Anti-dsDNA antibody (yes versus no)	1.48 (0.74–2.97)	.270		
Urine protein (per 1 g/24 h)	1.06 (0.98–1.15)	.138		
SCr (per 1 mg/dl increase)	1.73 (1.51–2.60)	.008	1.62 (1.04–2.54)	.035
AKI (yes versus no)	1.92 (0.89–4.16)	.098	0.87 (0.33–2.25)	.768
Leukocyturia (non-infection) (yes versus no)	1.93 (1.02–3.65)	.045	1.39 (0.63–3.06)	.417

ESR: erythrocyte sedimentation rate.

### Longitudinal association of haematuria with clinical endpoints

#### Kaplan–Meier survival estimates for haematuria-associated outcomes (***n*** = 178)

**Figure 2:**
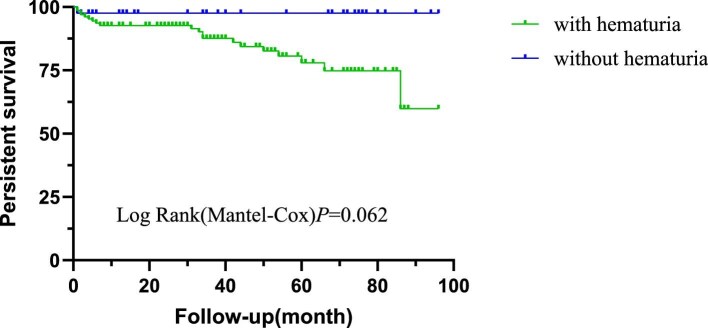
Kaplan–Meier analysis of composite outcomes between patients with and without haematuria at the initial diagnosis of LN.

**Figure 3:**
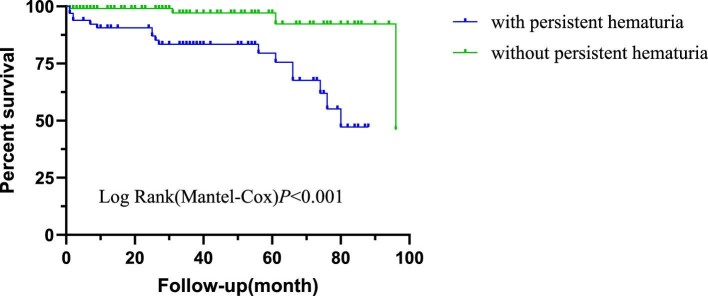
Kaplan–Meier analysis of composite outcomes for LN patients based on persistent haematuria during the remission of LN.

In the cohort, 21 of 178 patients (11.8%) experienced the composite outcome, including 9 deaths (42.9%), 6 ESRD (28.6%) and 6 with doubling of SCr (28.6%). Patients with haematuria have a tendency for a poor prognosis, with a median survival time of 80.48 months (95% CI 74.30–86.66) compared with 87.78 months (95% CI 83.47–92.08) in those without haematuria (logrank *P* = .062; shown in Fig. 2). However, further analysis revealed that persistent haematuria was a stronger predictor of adverse outcomes than the presence of haematuria at the initial diagnosis (*P* < .001; shown in Fig. 3).

#### Adjusted multivariable Cox regression modelling with time-dependent covariates: association between persistent haematuria and composite clinical endpoints (***n*** = 178)

In the full cohort analysis of 178 LN patients, we employed Cox proportional hazards models to evaluate the association between persistent haematuria and adverse outcomes (death or ESRD or doubling of SCr), with covariate adjustment strategies detailed in the Methods section. Schoenfeld residual testing revealed violation of the PH assumption for persistent haematuria stratification (*P* = .026), while all other variables satisfied the PH assumptions (*P* > .05). Pearson correlation analysis between covariate residuals and time ranks is presented in the supplementary materials (Supplementary Table S1). We therefore constructed a time-dependent Cox model incorporating time-varying covariates to adjust for temporal bias.

The time-dependent Cox regression demonstrated significant temporal variation in the effect of persistent haematuria on adverse outcomes, characterized by the time-varying coefficient equation: β(*t*) = −0.8 + 1.224 × ln(*t* + 1), with the corresponding hazard ratio (HR) formula: HR(*t*) = exp[−0.8 + 1.224 × ln(*t* + 1)]. The overall time-averaged effect of persistent haematuria yielded an HR of 3.40 (95% CI 1.06–10.90; *P* = .039), indicating a significantly elevated risk of adverse outcomes associated with persistent haematuria during follow-up. To quantify short-term risk, at the 12-month follow-up, β(12) = 2.339, corresponding to an HR of 10.37, demonstrating that patients with persistent haematuria had an 10.37-fold higher risk of adverse outcomes compared with non-persistent cases at this time point, reflecting a progressive risk elevation pattern (detailed results are presented in Table 5).

**Table 5:** Adjusted multivariable Cox regression modelling with time-dependent covariates: association between persistent haematuria and composite clinical endpoints.

	Bivariate analysis		Multivariable analysis		
Variables	HR (95% CI)	*P*-value	Adjusted HR (95% CI)	*P*-value	β
Age (per year)	1.03 (1.00–1.06)	.064	1.02 (0.99–1.06)	.166	0.024
SCR (per 1 mg/dl increase)	1.42 (1.25–1.61)	<.001	1.20 (0.97–1.48)	.090	0.181
Urine protein (per 1 g/24 h)	1.02 (0.91–1.15)	.738	0.97 (0.82–1.16)	.744	−0.029
C3 (per 1 g/l)	0.41 (0.06–2.93)	.373	1.24 (0.06–24.12)	.888	0.214
Haemoglobin (per 1 g/l)	0.98 (0.97–0.99)	.006	0.99 (0.97–1.01)	.345	−0.010
CRP (per 1 mg/l)	1.02 (1.01–1.04)	<.001	1.02 (1.00–1.04)	.016	0.019
Leukocyturia (non-infection) (yes versus no)	2.44 (1.03–5.75)	.042	1.63 (0.58–4.60)	.359	0.486
AKI (yes versus no)	3.26 (1.34–7.93)	.009	2.34 (0.62–8.94)	.212	0.852
Immunosuppressive regimens	0.93 (0.27–3.16)	.907	1.27 (0.22–7.23)	.785	0.242
Persistent haematuria (yes versus no)	8.83 (2.96–26.35)	<.001	0.45 (0.04–5.65)	.536	−0.800
Persistent haematuria (time-dependent variable)			3.40 (1.06–10.90)	.039	1.224

#### Stratified Cox proportional hazards analysis: association between persistent haematuria and renal outcomes stratified by pathological classification (***n***=103)

Cox proportional hazards models were employed to evaluate the association between persistent haematuria and adverse outcomes (death or ESRD or doubling of SCr), with covariate adjustment strategies detailed in the Methods section. The Schoenfeld residual test revealed violation of the PH assumption for pathological classification (*P* = .004), while all other variables satisfied the PH assumptions (*P* > .05). Consequently, pathological classification was incorporated as a stratified variable to control for potential bias. Pearson correlation analysis results between independent variable residuals and time ranks are presented in the supplementary materials (Supplementary Table S2). Multivariable analysis demonstrated that after covariate adjustment, persistent haematuria remained an independent risk factor for adverse outcomes [adjusted HR 9.32 (95% CI 1.05–82.65); *P* = .045] (displayed in Table 6).

**Table 6:** Stratified Cox proportional hazards analysis: association between persistent haematuria and renal outcomes stratified by pathological classification (*n* = 103).

	Bivariate analysis		Multivariable analysis	
Variables	HR (95% CI)	*P*-value	Adjusted HR (95% CI)	*P*-value
Age (per year)	1.05 (1.01–1.10)	.014	1.06 (1.02–1.10)	.005
SCr (per 1 mg/dl increase)	2.07 (1.36–3.14)	.001	1.85 (1.09–3.15)	.024
Urine protein (per 1 g/24 h)	1.05 (0.92–1.21)	.465	1.03 (0.89–1.20)	.677
Low C3 (yes versus no)	1.88 (0.24–14.74)	.546	0.22 (0.02–2.82)	.243
Pathological classification (proliferative versus non-proliferative)	51.11 (0.37–7074.52)	.118		
Persistent haematuria (yes versus no)	17.47 (2.22–137.23)	.792	9.32 (1.05–82.65)	.045

## DISCUSSION

Approximately one-third of LN patients who achieved complete clinical remission according to conventional criteria continue to exhibit histopathological activity upon subsequent renal biopsy [18]. This disconnect implies that standard clinical indicators, such as proteinuria and serological markers, may not adequately capture residual renal inflammation. Although repeat renal biopsy is considered the gold standard for evaluating disease activity, its invasive nature restricts its routine use in clinical practice [19]. These observations highlight that non-invasive biomarkers that can effectively identify ongoing glomerular and tubulointerstitial inflammation and are essential for enhancing long-term therapeutic approaches and prognostic assessments in LN.

The prognostic relevance of haematuria in renal diseases is well documented in the context of IgAN, with substantial evidence linking it to disease activity and adverse outcomes [20–22]. Notably, large cohort studies have shown that patients with persistent haematuria experience significantly higher rates of ESRD or a 50% decline in renal function compared with those with minimal or absent haematuria [23]. However, the clinical implications of persistent haematuria in LN have yet to be thoroughly investigated. This phenomenon is often categorized as a secondary symptom rather than being recognized as a distinct prognostic factor [24]. In our study, we found that 66 of 178 cases (37.1%) still present with persistent haematuria. This subgroup exhibited distinct clinical features, including elevated SLEDAI scores and increased SCr and BUN levels, alongside significantly lower haemoglobin, albumin and complement levels. Further, time-dependent Cox regression analysis further confirmed the association between persistent haematuria and adverse outcomes in LN: during the entire follow-up period, patients with persistent haematuria had a 3.40-fold increased time-averaged risk of adverse outcomes compared with those without persistent haematuria [HR 3.40 (95% CI 1.061–10.90), *P* = .039]. Notably, this risk effect was not fixed but showed an increasing trend over time—at 12 months of follow-up, the risk of adverse outcomes in patients with persistent haematuria had increased to 10.37-fold. These results not only challenge the traditional proteinuria-centric approach to monitoring LN but also suggest that haematuria may not merely be an epiphenomenon reflecting glomerular inflammation. Rather, it appears to be a clinically significant marker closely associated with an increased risk of adverse outcomes and poor long-term prognosis in LN patients.

Persistent haematuria may serve as a significant differentiator between ‘apparent remission’ and ‘true immunological quiescence’. This distinction is crucial for identifying high-risk patients who continue to experience subclinical nephron injury despite achieving control over proteinuria [25]. This notion aligns with emerging proposals to redefine complete renal remission in LN by integrating both the resolution of haematuria and the normalization of proteinuria [26]. Notably, while recent research has established the prognostic significance of stringent clinical remission as a treatment objective, our findings provide compelling evidence supporting the inclusion of haematuria assessment within this remission framework [13].

Current therapeutic approaches in LN predominantly focus on reducing proteinuria and utilizing immunosuppressive agents, with no evidence-based interventions specifically targeting haematuria [27]. Several critical questions require immediate exploration: Does haematuria in LN signify ongoing immune activation or complement-mediated injury? Are existing therapies, such as hydroxychloroquine, Blys blockade or complement inhibitors, capable of effectively alleviating haematuria? The limited systematic investigation into these inquiries likely arises from a historical underappreciation of the clinical relevance of haematuria. We advocate for the inclusion of haematuria as a secondary endpoint in future clinical trials to evaluate its therapeutic responsiveness and prognostic significance. Furthermore, research on combination therapies aimed at addressing haematuria is warranted, particularly in the context of developing effective management strategies for persistent haematuria.

It should be noted that this study primarily focuses on the association between persistent haematuria and clinical outcomes in LN without delving into its underlying biological mechanisms. Although existing studies suggest that haematuria may contribute to renal injury progression through multiple pathways, the precise role of erythrocyte-mediated kidney injury mechanisms in LN remains unclear and warrants further basic research and translational evidence [19, 28, 29]. Therefore, rather than elaborating on potential mechanisms, this discussion emphasizes the clinical utility of haematuria as a biomarker—regardless of its pathophysiological basis, its robust association with adverse outcomes appears sufficient to justify its inclusion in LN monitoring and remission assessment protocols.

This study has several limitations, including potential biases associated with the retrospective design, the lack of protocol-driven repeated renal biopsies (precluding clarification of the pathological basis of haematuria) and data on disease relapses (including both clinical relapses and those confirmed by biopsy). Future multicentre prospective studies that incorporate non-invasive biomarkers and repeat pathological evaluations are essential to further substantiate the role of haematuria in the management of LN. We further recommend complementary basic research to elucidate the precise mechanistic pathways through which haematuria contributes to LN progression.

## CONCLUSION

Persistent haematuria is an independent risk factor for adverse renal outcomes, indicating the need for further exploration. Continuous monitoring of haematuria in clinical practice, along with targeted intervention studies, is essential to improve long-term prognoses for patients with LN.

## Supplementary Material

sfaf348_Supplemental_File

## Data Availability

The data used and analysed in the current study are available from the corresponding author upon reasonable request.
