# Supplementary material for: Prognostic relevance of persistent haematuria in patients with lupus nephritis
Source: Clin Kidney J. 2025 Nov 13;19(1):sfaf348. doi: 10.1093/ckj/sfaf348 (PMC12771365; doi:10.1093/ckj/sfaf348)
Supplement: sfaf348_Supplemental_File [file sfaf348_supplemental_file.docx]

Table S1: Pearson correlation analysis between covariate residuals and time ranks for adjusted multivariable Cox regression models with time-dependent covariates(n=178)

|  | Pearson correlation coefficient | P value |
| --- | --- | --- |
| Age | -0.20 | 0.422 |
| Serum creatinine(umol/L) | 0.17 | 0.477 |
| Urine protein( 1g/24 h) | 0.24 | 0.322 |
| C3(1g/L) | 0.03 | 0.891 |
| Hemoglobin(g/L) | 0.34 | 0.158 |
| CRP(mg/L) | 0.04 | 0.859 |
| Leukocyturia (noninfection)(yes or no) | 0.03 | 0.897 |
| AKI(yes or no) | -0.21 | 0.393 |
| Immunosuppressive regimens | -0.19 | 0.434 |
| Persistent haematuria(yes or no) | 0.51 | 0.026 |

Notes: C3, Complement component 3; CRP, C-reactive protein; AKI, acute kidney injury.

Table S2: Pearson correlation analysis results between independent variable residuals and time ranks for stratified Cox proportional hazards models stratified by pathological classification(n=103).

|  | Pearson correlation coefficient | *P* value |
| --- | --- | --- |
| Age | -0.05 | 0.879 |
| Serum creatinine(umol/L) | 0.18 | 0.593 |
| Urine protein(g/24 h) | 0.20 | 0.559 |
| Low C3(yes or no) | -0.02 | 0.965 |
| Pathological classification (proliferative or non-proliferative) | 0.79 | 0.004 |
| Persistent haematuria(yes or no) | 0.32 | 0.333 |

Notes: C3, Complement component 3

Table S3 Haematuria characteristics and endpoint outcome by histopathological subtypes in biopsy-proven lupus nephritis(n=103).

|  | proliferative LN | non-proliferative LN | χ 2 | *P* |
| --- | --- | --- | --- | --- |
| Initial hematuria incidence(%) | 93.3% | 67.4% | 11.643 | 0.001 |
| Persistent hematuria incidence(%) | 53.3% | 20.9% | 10.976 | 0.001 |
| Endpoint outcome incidence(%) | 18.3% | 0% | 8.826 | 0.002 |
